# Evaluation of acute myeloid leukemia blast percentage on MethylC-Capture Sequencing results

**DOI:** 10.1186/s40164-021-00219-0

**Published:** 2021-03-31

**Authors:** Erna Yang, Desheng Gong, Wei Guan, Jieying Li, Xuefeng Gao, Yonghui Li, Li Yu

**Affiliations:** 1 Department of Hematology and Oncology, International Cancer Center, Shenzhen Key Laboratory of Precision Medicine for Hematological Malignancies, Shenzhen University General Hospital, Shenzhen University Clinical Medical Academy, Shenzhen University Health Science Center, Shenzhen University, Xueyuan AVE 1098, Nanshan District, Shenzhen, Guangdong 518000 People’s Republic of China; 2 Centrol Laboratory, Shenzhen University General Hospital, Shenzhen University Health Science Center, Xueyuan AVE 1098, Nanshan District, Shenzhen, Guangdong 518000 People’s Republic of China; 3 Department of Hematology, Chinese PLA General Hospital, Beijing, 100853 China

**Keywords:** AML, Blast percentage, Methylation status, MCC-Seq

## Abstract

Aberrant DNA methylation is often related to the diagnosis, prognosis, and therapeutic response of acute myeloid leukemia (AML); however, relevant studies on the relationship between bone marrow myeloblast percentage and the DNA methylation level in AML have not been reported. We evaluated the effects of AML blast percentage on DNA methylation level using the MethylC-capture sequencing (MCC-Seq) approach based on next-generation sequencing (NGS) and found that the methylation level of both genome-wide and promoter regions significantly increased when the percentage of AML blasts reached ≥ 40%, indicating that an accurate DNA methylation level in cancer cells can be obtained when the bone marrow samples of AML patients have more than 40% myeloblasts.

**Supplementary Information:**

The online version contains supplementary material available at 10.1186/s40164-021-00219-0.

To the editor,

Acute myeloid leukemia (AML) is a heterogeneous disease with the clonal disorder expansion of myeloid precursors and defined as ≥ 20% myeloblasts. A recent analysis on global data showed a continuously increasing trend of this disease in the past 28 years [1]. As a hallmark of AML, aberrant DNA methylation is often related to the diagnosis, prognosis, and therapeutic response. It was noted that cytogenetically defined AML subtypes have unique epigenetic signatures, and a DNA methylation classifier predicts the clinical outcome in AML [2, 3]. However, whether the diagnostic bone marrow (BM) myeloblast percentage has current value with regard to these next-generation sequencing (NGS) techniques is still unclear, and relevant studies on the relationship between myeloblast percentage and the DNA methylation level in AML patients have not been reported. We sought to evaluate the effects of acute leukemia blast percentage on the DNA methylation level. Among the current analysis techniques for DNA methylation, MethylC-capture sequencing (MCC-Seq) [4] based on NGS approach was developed for the targeted assessment of DNA methylation in a tissue-specific manner and successfully applied to targeting sperm epigenome [5–7].

Recently, we used MCC-Seq for detecting the genome-wide DNA methylation status of five healthy donors’ BM and an AML cell line (SKNO-1). The MCC-Seq assay was performed using the SeqCap Epi probe design platform of Roche NimbleGen. Each capture was sequenced on a single lane of 125 bp paired-end Illumina HiSeq2500 System. (Fig. 1). (The details of bioinformatics analysis are shown in Additional File 1). We evaluated the genome-wide DNA methylation original data and found that the original data quality is qualified without migration (Additional file 2: Table S1). Then, we analyzed the genome methylation status of BM samples from five healthy donors and SKNO-1 cells.

**Fig. 1:**
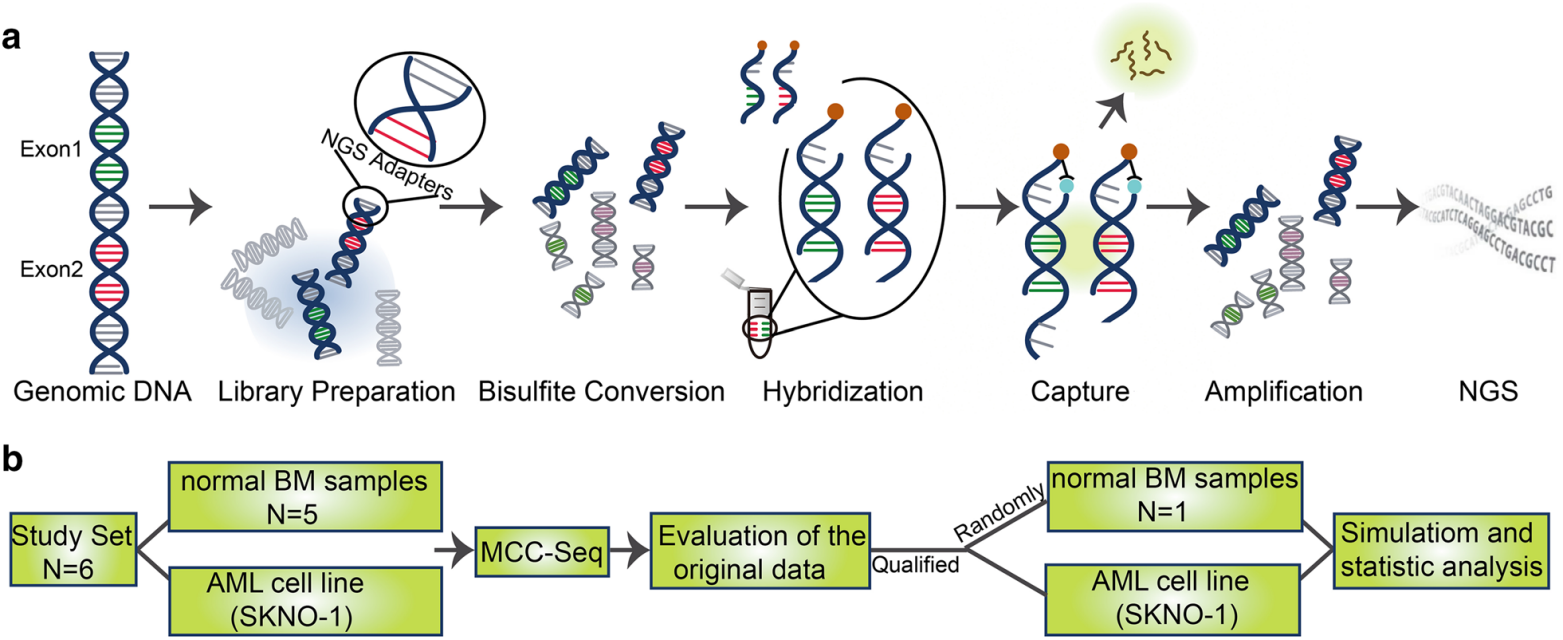
Brief workflow of this study. **a** Procedure for detecting the genome-wide DNA methylation with MCC-Seq; **b** Flowchart for experimental design and informatics analysis

The analysis results show that the DNA methylation levels are similar among the five healthy donors at both genome-wide (Fig. 2a) and promoter regions (TSS up 1 kb to down 0.1 kb) (Fig. 2b). The SKNO-1 cells had an apparent hypermethylation in contrast to the five healthy donors at both the whole genome and promoter regions (Fig. 2a, b). Furthermore, we randomly selected the sequences from a healthy donor and SKNO-1 cells, mixed them in a different ratio (from 0/10 to 10/0), and compared their methylation profiles (Additional file 3: Table S2). We found that the genomic methylation status of mixed cell population (simulated BM of AML patients) significantly increased (*P* < 0.01) when the percentage of SKNO-1 cells reached ≥ 40% (Fig. 2c). Notably, the genomic methylation level increased with the percentage of myeloblasts in the whole cell population. Moreover, the change in the methylation level of promoter regions showed a similar trend as the genome level (Fig. 2d). These results indicate that an accurate DNA methylation level in cancer cells can be obtained when the BM samples of AML patients have more than 40% myeloblasts (Additional file 4).

**Fig. 2:**
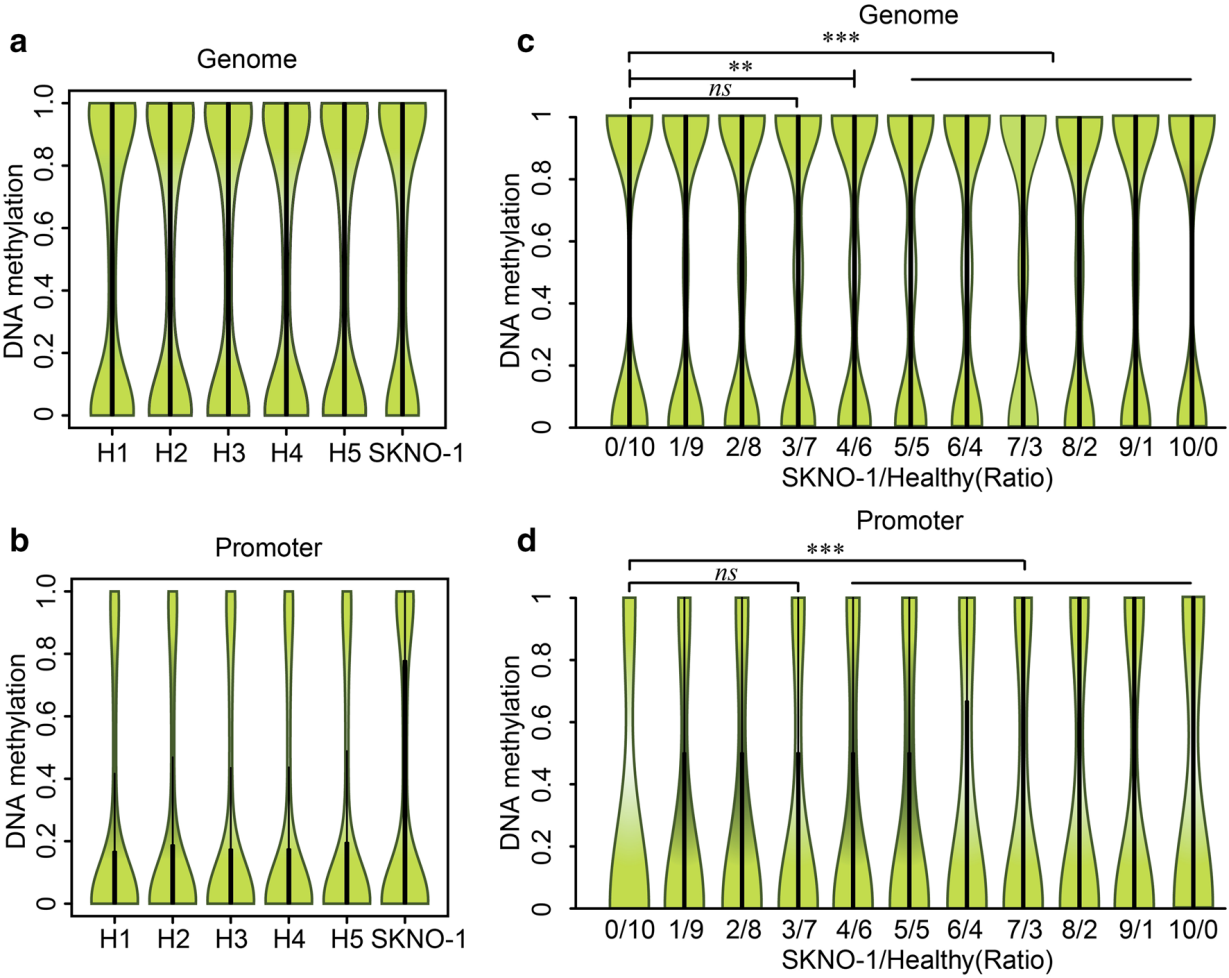
DNA methylation status with different acute myeloid blast percentages. **a** Genome methylation status of five healthy donors and SKNO-1 cells. **b** Promoter region (TSS up 1 kb to down0.1 kb) methylation status of five healthy donors and SKNO-1 cells. **c** Genome methylation status of different SKNO-1/ Healthy Ratio (from 0 to 100%). **d** Promoter region (TSS up 1 kb to down 0.1 kb) methylation status of different SKNO-1/ Healthy Ratio (from 0 to 100%). H, healthy donor; *ns*, no statistical difference; **, *P* < 0.01; ***, *P* < 0.001

The DNA methylation status has been assessed and confirmed to be a reliable and feasible part of clinical diagnosis, treatment, and prognosis of diseases, especially for those with high heterogeneous diseases such as AML [8]. As the aberrant DNA methylation profiles are a characteristic feature of AML, it raises interesting questions: Does BM myeloblast percentage reflect a different genome DNA methylation profile of AML or even different subtypes? In this study, we hypothesize that DNA methylation is distributed into specific patterns in AML with different myeloblast percentages, which may be associated with the response to hypomethylating agents (HMAs) and clinical outcomes, and we demonstrated this through the simulation BM of AML patients at different myeloblast percentages. So far, low-dose HMAs have been the mainstay for the treatment of higher-risk myelodysplastic syndrome (MDS) and also of elderly unfit AML patients [9, 10]. These data suggest that HMAs may not only inhibit DNA methylation, but also inhibit other molecular actions not related to DNA methylation [11]. Notably, methylation evaluation is more accurate when myeloblast percentage in the BM exceeds 40%. However, further validation based on clinical samples is necessary to confirm this cut-off ratio.

In conclusion, the percent of AML blasts in the BM affects the detected methylation level in AML patients, and this should be considered when making diagnosis and interpreting their clinical outcomes or response to chemotherapeutic agents.

## Supplementary Information

**Additional file 1**: Details for bioinformatics analysis.

**Additional file 2**: **Table S1**. The original data quality.

**Additional file 3**: **Table S2**. Data statistics randomly selected.

**Additional file 4**.

## Data Availability

The data and materials used and analyzed during the current study are available from the corresponding authors on reasonable request.

## Abbreviations

AML: Acute myeloid leukemia
BM: Bone marrow
MCC-Seq: MethylC-capture sequencing
MDS: Myelodysplastic syndrome
NGS: Next-generation sequencing
HMAs: Hypomethylating agents

## References

[1] Yi M, Zhou L, Li A, Luo S, Wu K (2020). Global burden and trend of acute lymphoblastic leukemia from 1990 to 2017. Aging.

[2] Figueroa ME, Lugthart S, Li Y, Erpelinck-Verschueren C, Deng X, Christos PJ (2010). DNA methylation signatures identify biologically distinct subtypes in acute myeloid leukemia. Cancer Cell.

[3] Yang X, Wong MPM, Ng RK (2019). Aberrant DNA methylation in acute myeloid leukemia and its clinical implications. Int J Mol Sci..

[4] Allum F, Shao X, Guenard F, Simon MM, Busche S, Caron M (2015). Characterization of functional methylomes by next-generation capture sequencing identifies novel disease-associated variants. Nat Commun.

[5] Chan D, Shao X, Dumargne MC, Aarabi M, Simon MM, Kwan T (2019). Customized MethylC-Capture sequencing to evaluate variation in the human sperm DNA methylome representative of altered folate metabolism. Environ Health Perspect.

[6] Allum F, Hedman ÅK, Shao X, Cheung WA, Vijay J, Guénard F (2019). Dissecting features of epigenetic variants underlying cardiometabolic risk using full-resolution epigenome profiling in regulatory elements. Nat Commun.

[7] Cao M, Shao X, Chan P, Cheung W, Kwan T, Pastinen T (2020). High-resolution analyses of human sperm dynamic methylome reveal thousands of novel age-related epigenetic alterations. Clin Epigenetics.

[8] Li Y, Xu Q, Lv N, Wang L, Zhao H, Wang X (2017). Clinical implications of genome-wide DNA methylation studies in acute myeloid leukemia. J Hematol Oncol.

[9] DiNardo CD, Pratz K, Pullarkat V, Jonas BA, Arellano M, Becker PS (2019). Venetoclax combined with decitabine or azacitidine in treatment-naive, elderly patients with acute myeloid leukemia. Blood.

[10] Wei AH, Strickland SA, Hou JZ, Fiedler W, Lin TL, Walter RB (2019). Venetoclax combined with low-dose cytarabine for previously untreated patients with acute myeloid leukemia: results from a phase Ib/II study. J Clin Oncol.

[11] Chiappinelli KB, Strissel PL, Desrichard A, Li H, Henke C, Akman B (2015). Inhibiting DNA methylation causes an interferon response in cancer via dsRNA including endogenous retroviruses. Cell.

