# Supplementary material for: Evaluation of acute myeloid leukemia blast percentage on MethylC-Capture Sequencing results
Source: Exp Hematol Oncol. 2021 Mar 31;10:26. doi: 10.1186/s40164-021-00219-0 (PMC8011402; doi:10.1186/s40164-021-00219-0)
Supplement: Supplementary file 1 — Additional file 1: Details for bioinformatics analysis. [file 40164_2021_219_MOESM1_ESM.docx]

**Details for bioinformatics analysis:**

Adapters were cut off from the raw reads, and then trimmed for quality (phred33≥30) and length (n≥50) using Perl script (FQ_clean_v2.0.pl, parameter: -q 5 -G 20 -L 50 -r 0.5 -N 0.1 -P 33). The filtered reads were aligned to the hg19 human reference using BSMAP (v 2.73, parameters: -*v* 0.1 -*g* 1 -*p* 8 -R -*u*) (*Xi Y, Li W. BSMAP: whole genome bisulfite sequence MAPping program. BMC bioinformatics.2009;10:232.*). Methylation calls were extracted based on the unique sequences. In all subsequent analyses, a sequence depth of ≥5X was used to detect the methylation level of CpGs. Metilene (v 0.2-6) (*Jühling F, Kretzmer H, Bernhart SH, Otto C, Stadler PF, Hoffmann S. metilene: fast and sensitive calling of differentially methylated regions from bisulfite sequencing data. Genome research. 2016;26(2):256-62.*) was employed to identify maximal between-group methylation differences in genomic regions of minimum length in combination with Mann-Whitney-U test. Results were further refined by selecting DMRs meeting the criteria: (1) minimum of five CpG sites, (2) maximum CpG distance (default:300), (3) minimum CpGs: 5, (4) minimum methylation difference (D-absolute value) ≥ 0.2, and (5) adjusted *P*< 0.05.
